# High rate of missed Barrett’s esophagus when screening with forceps biopsies

**DOI:** 10.1007/s10388-022-00943-4

**Published:** 2022-07-22

**Authors:** Mendel E. Singer, Robert D. Odze

**Affiliations:** 1 Department of Population and Quantitative Health Sciences, School of Medicine, Case Western Reserve University, 10900 Euclid Ave, WOM WG-57, Cleveland, OH 44106 USA; 2 Department of Pathology and Lab Medicine, Tufts University School of Medicine, Boston, MA USA

**Keywords:** Barrett’s esophagus, Esophageal cancer, Endoscopy, Epidemiology, Test sensitivity

## Abstract

**Background:**

Screening for Barrett’s esophagus (BE) with endoscopy plus forceps biopsy (FB) has poor compliance with the recommended Seattle protocol and fails to sample large areas of mucosa. This statistical modeling study estimates, for the first time, the actual frequency of missed BE cases by FB.

**Methods:**

Published, calibrated models in the literature were combined to calculate the age-specific prevalence of BE in white males with gastroesophageal reflux disease (GERD). We started with estimates of the prevalence of BE and GERD, and applied the relative risk for BE in patients with GERD based on the literature. This created estimates of the true prevalence of BE in white males with GERD by decade of life. The proportion of BE missed was calculated as the difference between the prevalence and the proportion with a positive screen.

**Results:**

The prevalence of BE in white males with GERD was 8.9%, 12.1%, 15.3%, 18.7% and 22.0% for the third through eighth decades of life. Even after assuming no false positives, missed cases of BE were about 50% when estimated for patients of ages 50 or 60 years, and over 60% for ages of 30, 40 or 70 years. Sensitivity analysis was done for all variables in the model calculations. For ages 50 and 60 years, this resulted in values from 30.3 to 57.3% and 36.4 to 60.9%.

**Conclusion:**

Screening for BE with endoscopy and FB misses approximately 50% of BE cases. More sensitive methods of BE detection or better adherence to the Seattle protocol are needed.

## Introduction

Barrett’s esophagus (BE) is a pre-cancerous condition understood to be the result of chronic inflammation due to gastroesophageal reflux disease (GERD) [1]. It is the only known precursor of esophageal adenocarcinoma (EAC). The Incidence of EAC has more than doubled from 1984 to 2013, with 5-year survival rates about 20% [2]. Studies have shown that patients with Barrett’s esophagus (BE) have a 30–60-fold increased risk of esophageal adenocarcinoma (EAC) [3]. Accordingly, guidelines recommend screening for BE, according to the Seattle protocol, in high risk populations, such as white males over the age of 50 with gastroesophageal reflux disease (GERD) [4–6]. The Seattle protocol consists of four quadrant forceps biopsies (FB) from every 1–2 cm of esophageal columnar mucosa combined with targeted biopsies of any visually identified suspicious lesions.

The sensitivity of FB for detection of BE (columnar mucosa with intestinal metaplasia) is unknown due to the lack of a superior gold standard. Use of forceps biopsies has been postulated to result in missed cases of BE due to only sampling small amounts of tissue, and lack of adherence to the Seattle protocol, among other reasons [7, 8]. However, the specific rate, or even magnitude, of misses has never been determined. A high miss rate would argue for interventions to improve adherence with protocols, or improved methods or technologies. Given the success of radiofrequency ablation for treating dysplastic BE [9], there is greater incentive to identify and surveil BE cases. This study employs a novel and simple approach that leverages existing published literature to calculate an estimate of the proportion of BE cases that are missed when screening with endoscopy and FB in white males with gastroesophageal reflux disease (GERD).

## Methods

The prevalence of BE in white males with GERD was compared to the results of FB screening in the published literature. We then calculated the miss rate by dividing the percentage of positive screens by the prevalence of BE, and then subtracting from 100%. This was performed for patients 30–70 years of age, at 10-year intervals. When the data were reported only by decade of life, estimates at a specific age were computed as an average (e.g., age 30 year estimates were based on the average of the 20–29 and 30–39 year old age groups).

Population-based screening results for white males with GERD by age were obtained from Rubenstein et al. [10]. For specific ages, we extracted data points from the graph using Engauge Digitizer V12.1 [11].

To estimate the true prevalence of BE, we solved two equations with two unknowns. The prevalence of BE is the sum of the marginal probabilities of BE in GERD and non-GERD patients:

$${\text{BE}}\,{\text{prevalence}}\, = \left( {prob\left( {{\text{GERD}}} \right)*{\text{BE}}\,{\text{prevalence}}\,{\text{in}}\,{\text{GERD}}} \right) + \left( {prob\left( {\text{Non - GERD}} \right)*{\text{BE}}\,{\text{prevalence}}\,{\text{in}}\,{\text{non-GERD}}} \right)$$

The relative risk (RR) of BE in GERD patients is:

$${\text{RR}}\,{\text{of}}\,{\text{BE}}\,{\text{in}}\,{\text{GERD}} = {{{\text{prevalence}}\,\,{\text{of}}\,{\text{BE}}\,{\text{in}}\,{\text{GERD}}} \mathord{\left/ {\vphantom {{{\text{prevalence}}\,\,{\text{of}}\,{\text{BE}}\,{\text{in}}\,{\text{GERD}}} {{\text{prevalence}}\,\,{\text{of}}\,{\text{BE}}\,{\text{in}}\,{\text{non - GERD}}}}} \right. \kern-\nulldelimiterspace} {{\text{prevalence}}\,\,{\text{of}}\,{\text{BE}}\,{\text{in}}\,{\text{non - GERD}}}}$$

**Fig. 1:**
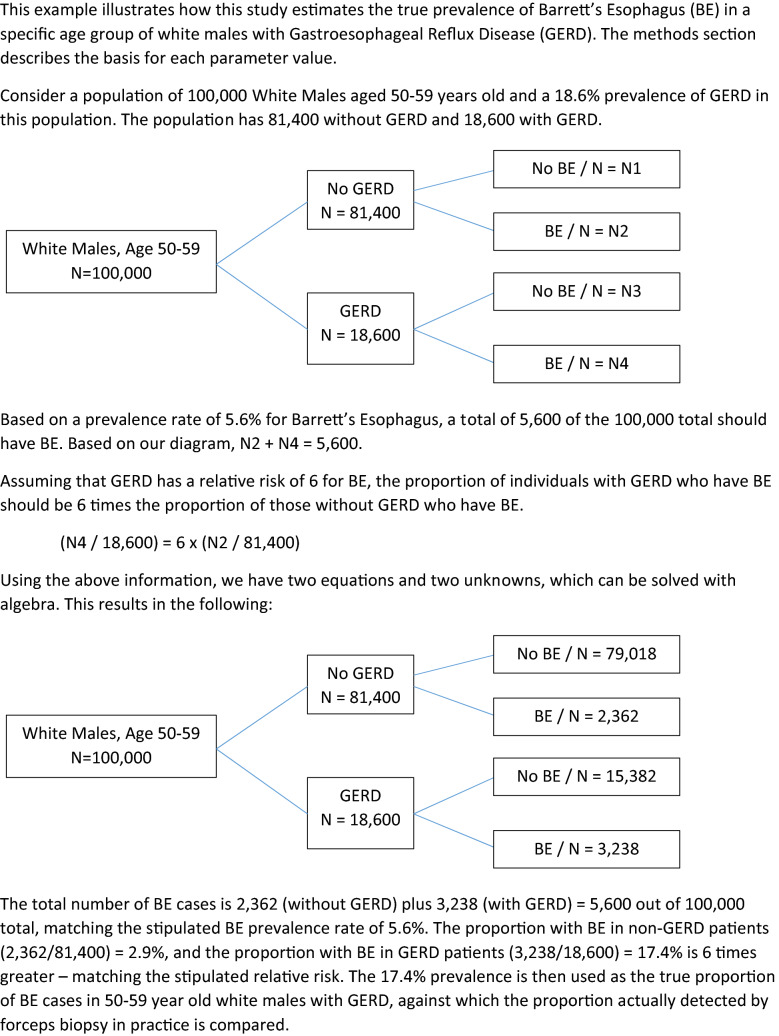
How the true prevalence of Barrett’s esophagus is estimated

These two equations have five unknown variables. However, literature is available to estimate three: prevalence of BE, prevalence of GERD and the RR for BE in patients with GERD. Given these three parameters, there are two simultaneous equations and two unknowns to solve for: prevalence of BE in white males with and without GERD. Figure 1 provides an illustration of our method.

### Estimation of prevalence of GERD and BE in the population

Hur et al. in their simulation model of EAC, required age-specific prevalence rates of GERD in the community [12]. These investigators performed a weighted regression analysis of 12 studies to determine an overall GERD prevalence rate of 18.6%. They also categorized the prevalence rate by decade of life. These were the estimates used. For sensitivity analysis, we varied the population prevalence rate of GERD from 16 to 22% based on a previously published 95% confidence interval, but we allowed for a lower value to be conservative [13].

Estimates of BE prevalence have varied widely. For instance, two widely cited studies estimated this value from 1.6 to 6.8% [14, 15]. The National Cancer Institute has sponsored model development for many types of common cancers, one of them being esophageal cancer. One of those models was developed by a team from Erasmus University Medical Center and the University of Washington (ERASMUS/UW). Their computer simulation disease model of EAC was calibrated against Surveillance, Epidemiology, and End Results (SEER) cancer incidence data [16]. The model estimated the true prevalence rate of BE in the population to be 5.6%. In the Hur et al. study quoted above where they used a breakdown of BE prevalence by decade of age, a midpoint of this range, 4.2%, was used. For our age-specific BE prevalence rates, we recalibrated the results of the study by Hur et al. to reflect this improved estimate of the BE prevalence rate in the population. Thus, the relative prevalence rate by decade of age was preserved. For our sensitivity analysis, we considered a range of 4.2–6.8%, and then scaled the age-specific prevalence rates accordingly.

### Calculation of relative risk (RR) of GERD

An essential parameter in our analysis is calculation of the relative risk (RR) of BE in GERD patients, which is required to convert BE prevalence in the white male population to the corresponding rate in white males with GERD. We examined estimates of the RR of BE in GERD patients from multiple sources: modeling studies, population-based studies and systematic reviews/meta-analyses.

In the ERASMUS/UW model, a relative risk for BE of 6 in patients with GERD was used [17, 18]. This was based on an estimate that 60% of BE patients have GERD (3:2 ratio), despite a prevalence rate of GERD of only 20% (1:4 ratio), and the RR representing a division of these two values, ${\text{RR}} = {{\left( {{3 \mathord{\left/ {\vphantom {3 2}} \right. \kern-\nulldelimiterspace} 2}} \right)} \mathord{\left/ {\vphantom {{\left( {{3 \mathord{\left/ {\vphantom {3 2}} \right. \kern-\nulldelimiterspace} 2}} \right)} {\left( {{1 \mathord{\left/ {\vphantom {1 4}} \right. \kern-\nulldelimiterspace} 4}} \right)}}} \right. \kern-\nulldelimiterspace} {\left( {{1 \mathord{\left/ {\vphantom {1 4}} \right. \kern-\nulldelimiterspace} 4}} \right)}} = 6$

A study that used the General Practice Research Database (GPRD) of about 3 million patients across 398 general practitioner practices in England and Wales, estimated GERD and BE incidence and prevalence over a 10 year period [19]. About 45% of incident BE cases were in patients with a documented diagnosis of GERD and the annual prevalence of GERD was 6.2%. This produces a RR of BE in GERD of 12.9, which is likely an overestimate due to undercounting of patients who visit their physician less than once per year.

A recent systematic review of the prevalence of BE can also be used to estimate the RR of BE in GERD [20]. Depending on the definition of BE, the RR of BE in GERD was 3.4, 7.0 or 14.3.

Further support for a high RR is found by Lieberman et al. who showed that patients with GERD symptoms for more than 10 years had an odds ratio for BE of 6.4 compared to patients with GERD symptoms of less than one year duration [21]. Since the prevalence in the latter group is relatively small, the relative risk would only be slightly less than the odds ratio.

In contrast, there are also sources that suggest a low RR. For instance, in one retrospective cohort study, by Jung et al. 0.22% of patients were diagnosed with BE over a 7–8 year follow-up period [22]. The odds ratio for BE in GERD patients was 3.67. Given the extremely low incidence of 0.22%, the RR would be virtually identical to the odds ratio.

A meta-analysis by Taylor et al. solved the problem of heterogeneity across studies by only including studies that invited the general population to participate, and then stratifying into short-segment BE (SSBE) and long-segment BE (LSBE) [23]. These authors arrived at an odds ratio of 4.92 for LSBE and 1.15 for SSBE. However, studies were mixed in their definition of LSBE, using either 2 cm or 3 cm. A recent study documented 1,061 of 1,883 BE cases ≥ 3 cm [24]. Using this prevalence of LSBE, the overall OR is 3.3. While the relative risk would be slightly lower, this is counterbalanced using the lower prevalence for 3 cm LSBE when the ORs were based on a mix of the more prevalent 2 cm as well as 3 cm LSBE.

For the main analysis, we used a RR of 6. In sensitivity analysis, we used a range of 3.3 to 9.0.

Model parameters are summarized in Table 1. All calculations were performed in Microsoft Excel 2016 (Microsoft Corp., Redmond WA, USA).

**Table 1:** Parameter values

		Sensitivity analysis	
	Value	Low	High
BE Prevalence	5.6%	4.2%	6.8%
Age 20–29	2.3%	Proportional to Change in Overall Prevalence	
Age 30–39	3.3%		
Age 40–49	4.4%		
Age 50–59	5.5%		
Age 60–69	6.7%		
Age 70–79	7.7%		
GERD prevalence	18.6%	16.0%	22.0%
Age 20–29	17.6%	Proportional to Change in Overall Prevalence	
Age 30–39	18.0%		
Age 40–49	18.4%		
Age 50–59	18.8%		
Age 60–69	19.1%		
Age 70–79	19.5%		
Relative risk for BE (in GERD patients)	6.0	3.3	9.0

## Results

The prevalence of BE in white males with GERD ranged from 7.2% for patients age 20–29 years, to 23.5% for ages 70–79 years, compared to 1.2% and 3.9% in non-GERD patients. The estimates at ages 30, 40, 50, 60 and 70 were determined by averaging, e.g., age 30 estimate was the average for ages 20–29 and 30–39. For ages 30, 40, 50, 60 and 70 years, the prevalence of BE in white males with GERD was 8.9%, 12.1%, 15.3%, 18.7% and 22.0%, respectively. (Table 2) Assuming no false positives, the rate of missed BE by age was 68.8%, 60.1%, 48.2%, 52.6% and 62.1%. However, the average for patients 50 and 60 years old, the most common and routine ages that patients are screened, was 50.4%.

**Table 2:** Forceps biopsy: sensitivity and missed cases of Barrett’s esophagus in white males with GERD

			%BE missed	
Age	BE prevalence (%)	Forceps biopsy positive (%)	Base case (%)	Sensitivity analysis (%)
30	8.9	2.8	68.8	57.7–73.3
40	12.1	4.8	60.1	46.2–67.2
50	15.3	7.9	48.2	30.3–57.3
60	18.7	8.9	52.6	36.4–60.9
70	22.0	8.3	62.1	49.3–68.7

In sensitivity analysis, results were affected more by the range of plausible values for BE prevalence and RR of BE, than for the GERD prevalence (Table 2). The range of test sensitivity values ranged from 42.7 to 69.7% for age 50 and 39.1–63.6% for age 60.

## Discussion

This is the first study to estimate the miss rate of diagnosing BE utilizing FB. We showed that screening for BE with endoscopy and FB misses approximately 50% of all BE cases, even after assuming all positive tests are true positives. Although a high miss rate with FB has always been suspected clinically, this particular modeling study is the first to demonstrate the magnitude of false negatives with FB. The results are not surprising considering the failure of FB to sample large areas of mucosa [8] and overall lack of adherence to the Seattle protocol. Adherence to the Seattle protocol was estimated to be 51.2% in a retrospective study by Abrams et al. in 2009 [7]. This was in the middle of the range found in survey-based studies [25–30], as well as in two more recent studies [31, 32]. There are other reasons why use of the Seattle protocol with FB might have poor sensitivity: low inter-observer agreement in diagnosing dysplasia among pathologists and high missed esophageal cancer rate at index endoscopy of BE patients [33, 34].

One question that is often asked is whether missed BE cases have a similar prognosis as those detected by FB. The prevalence estimate of BE used in this modeling study was obtained from a model of esophageal cancer (EAC) that calibrated BE prevalence rates such that the resulting EACs would align with known rates from SEER data [16]. However, if missed cases of BE progress at a slower rate than those detected by FB, it would require an even larger BE prevalence rate than assumed here to produce the same number of cancers. This would result in the BE miss rate for FB being even higher than that reported here.

Our model used three main parameters. The prevalence data of BE and GERD were obtained from well- designed and calibrated models, but the RR of BE in patients with GERD was, admittedly, difficult to estimate. For instance, screening studies have used widely variable definitions of GERD, and the definition of BE has also varied over time and by geographic region. Nevertheless, sensitivity analysis showed that even by decreasing the RR by nearly half, there was a high rate of missed BE cases.

The population database used in this study included some unknown number of patients evaluated for GERD who previously had BE ruled out at a different clinical practice and were not biopsied. This results in a lower proportion of positive screens, a likely small bias against FB. This should be outweighed by our assumption of no false positives, a possibly large bias in favor of FB.

Studies of BE surveillance have shown that length of BE is associated with worse adherence with the Seattle protocol and a higher rate of missed dysplasia [7, 31]. However, it is unknown if poor adherence to the Seattle protocol is related to longer length of esophageal columnar mucosa in screening. Unfortunately, this study cannot address this question as it lacked information regarding length of mucosa or BE.

Surveillance of BE has been shown to be cost effective, and leads to earlier diagnosis of EAC and improved survival [35–38]. Radiofrequency ablation provides long-lasting success for treatment of dysplasia, thereby preventing EAC [9]. However, these benefits cannot be properly realized in the absence of a highly sensitive screening method for BE. A review by Chandar et al. [39] emphasized newer, less invasive methods. A 2019 review by Steele et al. of 18 evolving technologies for screening and surveillance of BE mentions four with increased sensitivity [40], only one of which is both available and shown to increase sensitivity in screening. Wide area trans-epithelial sampling with computer-assisted 3-dimensional analysis (WATS3D), when added to FB, has been shown in large community studies to increase the detection of BE by 83% and 153%, respectively — consistent with the findings in this study [41, 42]. A small, recent study suggests that progression to high-grade dysplasia or cancer may be similar for the additional BE cases detected by WATS and those identified by FB [8, 43].

In summary, we have presented, for the first time, statistical evidence that FB may miss approximately half of all BE cases during screening. Improvement might result from greater adherence to the Seattle protocol and/or with application of newer, more sensitive methods or technology for BE detection.

## References

[1] Phillips WA, Lord RV, Nancarrow DJ (2011). Barrett’s esophagus. J Gastroenterol Hepatol.

[2] Haiyu Z, Xiaofeng P, Xiangqiong M (2019). Incidence and survival changes in patients with esophageal adenocarcinoma during 1984–2013. Biomed Res Int.

[3] Lagergren J (2005). Adenocarcinoma of oesophagus: what exactly is the size of the problem and who is at risk?. Gut.

[4] Shaheen NJ, Falk GW, Iyer PG (2016). ACG Clinical Guideline: diagnosis and management of Barrett’s esophagus. Am J Gastroenterol.

[5] Qumseya B, Sultan S, ASGE Standards Of Practice Committee (2019). ASGE guideline on screening and surveillance of Barrett’s esophagus. Gastrointest Endosc.

[6] Spechler SJ, Sharma P, Souza RF (2011). American Gastroenterological Association medical position statement on the management of Barrett’s esophagus. Gastroenterology.

[7] Abrams JA, Kapel RC, Lindberg GM (2009). Adherence to biopsy guidelines for Barrett’s esophagus surveillance in the community setting in the United States. Clin Gastroenterol Hepatol.

[8] Codipilly DC, Iyer PG (2022). What’s next for wide-area transepithelial sampling in Barrett’s esophagus management?. Gastrointest Endosc.

[9] Orman ES, Li N, Shaheen NJ (2013). Efficacy and durability of radiofrequency ablation for Barrett’s esophagus: systematic review and meta-analysis. Clin Gastroenterol Hepatol.

[10] Rubenstein JH, Mattek N, Eisen G (2010). Age- and sex-specific yield of Barrett’s esophagus by endoscopy indication. Gastrointest Endosc.

[11] Mark M. BMaTWea. Engauge Digitizer Software http://markummitchell.github.io/engauge-digitizer/ (2021). Accessed 2 Sept 2021

[12] Hur C, Hayeck TJ, Yeh JM (2010). Development, calibration, and validation of a US white male population-based simulation model of esophageal adenocarcinoma. PLoS ONE.

[13] Locke GR, Talley NJ, Fett SL (1997). Prevalence and clinical spectrum of gastroesophageal reflux: a population-based study in Olmsted County Minnesota. Gastroenterology.

[14] Rex DK, Cummings OW, Shaw M (2003). Screening for Barrett’s esophagus in colonoscopy patients with and without heartburn. Gastroenterology.

[15] Ronkainen J, Aro P, Storskrubb T (2005). Prevalence of Barrett’s esophagus in the general population: an endoscopic study. Gastroenterology.

[16] Hayeck TJ, Kong CY, Spechler SJ (2010). The prevalence of Barrett’s esophagus in the US: estimates from a simulation model confirmed by SEER data. Dis Esophagus.

[17] Kroep S, Lansdorp-Vogelaar I, Rubenstein JH (2015). An accurate cancer incidence in Barrett’s esophagus: a best estimate using published data and modeling. Gastroenterology.

[18] Kroep S, Lansdorp-Vogelaar I, van der Steen A (2015). The impact of uncertainty in Barrett’s esophagus progression rates on hypothetical screening and treatment decisions. Med Decis Making.

[19] Alexandropoulou K, van Vlymen J, Reid F (2013). Temporal trends of Barrett’s oesophagus and gastro-oesophageal reflux and related oesophageal cancer over a 10-year period in England and Wales and associated proton pump inhibitor and H2RA prescriptions: a GPRD study. Eur J Gastroenterol Hepatol.

[20] de Sa Marques I, Marcos P, Sharma P (2020). The global prevalence of Barrett’s esophagus: a systematic review of the published literature. United European Gastroenterol J.

[21] Lieberman DA, Oehlke M, Helfand M (1997). Risk factors for Barrett's esophagus in community-based practice. GORGE consortium. Gastroenterology outcomes research group in endoscopy. Am J Gastroenterol.

[22] Jung KW, Talley NJ, Romero Y (2011). Epidemiology and natural history of intestinal metaplasia of the gastroesophageal junction and Barrett’s esophagus: a population-based study. Am J Gastroenterol.

[23] Taylor JB, Rubenstein JH (2010). Meta-analyses of the effect of symptoms of gastroesophageal reflux on the risk of Barrett’s esophagus. Am J Gastroenterol.

[24] Hamade N, Vennelaganti S, Parasa S (2019). Lower annual rate of progression of short-segment vs long-segment Barrett’s Esophagus to esophageal adenocarcinoma. Clin Gastroenterol Hepatol.

[25] Gross CP, Canto MI, Hixson J (1999). Management of Barrett’s esophagus: a national study of practice patterns and their cost implications. Am J Gastroenterol.

[26] van Sandick JW, Bartelsman JF, van Lanschot JJ (2000). Surveillance of Barrett’s oesophagus: physicians’ practices and review of current guidelines. Eur J Gastroenterol Hepatol.

[27] Das D, Ishaq S, Harrison R (2008). Management of Barrett’s esophagus in the UK: overtreated and underbiopsied but improved by the introduction of a national randomized trial. Am J Gastroenterol.

[28] Falk GW, Ours TM, Richter JE (2000). Practice patterns for surveillance of Barrett’s esophagus in the united states. Gastrointest Endosc.

[29] Ofman JJ, Shaheen NJ, Desai AA (2001). The quality of care in Barrett’s esophagus: endoscopist and pathologist practices. Am J Gastroenterol.

[30] Menezes A, Tierney A, Yang YX (2015). Adherence to the 2011 American Gastroenterological Association medical position statement for the diagnosis and management of Barrett’s esophagus. Dis Esophagus.

[31] Wani S, Williams JL, Komanduri S (2019). Endoscopists systematically undersample patients with long-segment Barrett’s esophagus: an analysis of biopsy sampling practices from a quality improvement registry. Gastrointest Endosc.

[32] Westerveld D, Khullar V, Mramba L (2018). Adherence to quality indicators and surveillance guidelines in the management of Barrett's esophagus: a retrospective analysis. Endosc Int Open.

[33] Kerkhof M, van Dekken H, Steyerberg EW (2007). Grading of dysplasia in Barrett’s oesophagus: substantial interobserver variation between general and gastrointestinal pathologists. Histopathology.

[34] Visrodia K, Singh S, Krishnamoorthi R (2016). Magnitude of missed esophageal adenocarcinoma after Barrett’s esophagus diagnosis: a systematic review and meta-analysis. Gastroenterology.

[35] Kroep S, Heberle CR, Curtius K (2017). Radiofrequency ablation of Barrett's esophagus reduces esophageal adenocarcinoma incidence and mortality in a comparative modeling analysis. Clin Gastroenterol Hepatol.

[36] Qiao Y, Hyder A, Bae SJ (2015). Surveillance in patients with Barrett’s esophagus for early detection of esophageal adenocarcinoma: a systematic review and meta-analysis. Clin Transl Gastroenterol.

[37] El-Serag HB, Naik AD, Duan Z (2016). Surveillance endoscopy is associated with improved outcomes of oesophageal adenocarcinoma detected in patients with Barrett’s oesophagus. Gut.

[38] Verbeek RE, Leenders M, Ten Kate FJ (2014). Surveillance of Barrett’s esophagus and mortality from esophageal adenocarcinoma: a population-based cohort study. Am J Gastroenterol.

[39] Krishna Chandar A, Sharma A, Chak A (2020). Novel Screening alternatives for Barrett esophagus. Gastroenterol Hepatol.

[40] Steele D, Baig KKK, Peter S (2019). Evolving screening and surveillance techniques for Barrett’s esophagus. World J Gastroenterol.

[41] Gross SA, Smith MS, Kaul V (2018). Increased detection of Barrett’s esophagus and esophageal dysplasia with adjunctive use of wide-area transepithelial sample with three-dimensional computer-assisted analysis (WATS). United European Gastroenterol J.

[42] Smith MS, Ikonomi E, Bhuta R (2019). Wide-area transepithelial sampling with computer-assisted 3-dimensional analysis (WATS) markedly improves detection of esophageal dysplasia and Barrett’s esophagus: analysis from a prospective multicenter community-based study. Dis Esophagus.

[43] Shaheen NJ, Smith MS, Odze RD (2022). Progression of Barrett’s esophagus, crypt dysplasia, and low-grade dysplasia diagnosed by wide-area transepithelial sampling with 3-dimensional computer-assisted analysis: a retrospective analysis. Gastrointest Endosc.

